# Contemporary Approaches to the Study of Cognitive Aging

**DOI:** 10.1093/geroni/igaa057.1913

**Published:** 2020-12-16

**Authors:** Gizem Hueluer, Elizabeth A L Stine-Morrow

## Abstract

Cognitive aging research is gaining societal and practical importance because of population aging. Current research is focused on describing age differences and age-related changes in cognitive performance, understanding potential causes underlying these differences and changes, and identifying factors that promote maintenance of cognitive functioning in old age. The goal of this research group is to showcase new developments in research studying age differences in cognitive performance and longitudinal cognitive change in the second half of life. Hülür et al. examine associations between midlife occupational factors and trajectories of cognitive change using data from the German Interdisciplinary Longitudinal Study of Adult Development and Aging (ILSE). Luo et al. use 12-year longitudinal data from 499 older participants in ILSE to study bidirectional associations between social relationships and cognitive performance. Small et al. examine the correspondence between objective and subjective cognitive performance, and measures of fatigue and depressed mood in experience sampling data from breast cancer survivors. Haas et al. compare laboratory and at-home online assessments of cognitive status and prospective memory over the adult lifespan and evaluate the quality of self-administered tests. The discussion by Elizabeth Stine-Morrow will focus on how these approaches contribute to our understanding of processes of cognitive aging and how they can be utilized to promote maintenance of cognitive functioning in old age.

